# Effects of *Eucommia ulmoides* Leaf-*Astragalus membranaceus* Complex on Antioxidant Capacity, Immune Function, Gut Microbiota and Serum Metabolome of Yellow-Feather Broilers

**DOI:** 10.3390/vetsci13101044

**Published:** 2026-10-05

**Authors:** Hongyan Ma, Yi Yao, Yanming Wei

**Affiliations:** 1 College of Veterinary Medicine, Gansu Agricultural University, Lanzhou 730070, China; mhy8110@163.com; 2 Department of Animal Science, Shanxi Yuncheng Agricultural Vocational and Technical College, Yuncheng 044000, China; yao_yige@126.com

**Keywords:** *Eucommia ulmoides* leaf, *Astragalus membranaceus*, herbal feed additive, yellow-feathered broiler, growth performance, gut microbiota, serum metabolomics, antioxidant capacity

## Abstract

**Background:** Phytogenic feed additives may provide sustainable alternatives for improving poultry productivity and health. This study evaluated the chemical composition, antioxidant activity, and in vivo effects of a 1:1 *Eucommia ulmoides* leaf–*Astragalus membranaceus* complex (EG) in yellow-feathered broilers. **Methods:** The nutritional composition, bioactive compounds, and antioxidant activity of EG were characterized. A total of 200 one-day-old yellow-feathered broilers were randomly allocated into four dietary treatments with five replicates per group and 10 birds per replicate: the control group (CG) receiving a basal diet and three EG supplementation groups receiving diets supplemented with 0.5% (EG (0.5%)), 1.0% (EG (1.0%)), 2.0% (EG (2.0%)) *Eucommia ulmoides* leaf-*Astragalus membranaceus* complex for 42 days. Growth performance, serum antioxidant and immune indicators, intestinal microbiota composition, and serum metabolomic profiles were analyzed. **Results:** EG contained 46.53% polysaccharides and exhibited concentration-dependent radical-scavenging activity. Supplementation with EG (0.5%) consistently increased body weight and average daily gain and reduced the feed conversion ratio without affecting feed intake. EG increased serum antioxidant enzyme activities and immunoglobulin concentrations but did not affect routine biochemical parameters. The EG (0.5%) group was therefore selected for microbiome and metabolome analyses. EG altered jejunal and cecal microbial composition without significantly affecting α-diversity and increased cecal *Faecalibacterium* and *Blautia* at day 21 and *Faecalibacterium* and *Parabacteroides* at day 42. Metabolomics identified 103 differential metabolites associated mainly with amino acid, glycerophospholipid, and energy metabolism. Spearman’s analysis further associated *Bacteroidota*, *Bacillota*, *Enterococcus*, and *Blautia* with amino acid, carbohydrate, and lipid-related metabolites. **Conclusions:** These findings indicate that EG (0.5%) may improve growth and feed efficiency while modulating antioxidant status, humoral immune indices, intestinal microbial composition, and systemic metabolism in yellow-feathered broilers.

## Simple Summary

Natural plant-based feed additives may help poultry producers improve growth and bird health while reducing reliance on antibiotic growth promoters. This study examined a 1:1 mixture of *Eucommia ulmoides* leaf and *Astragalus membranaceus* complex in yellow-feathered broilers. The mixture contained abundant polysaccharides and showed antioxidant activity in laboratory tests. When added to feed for 42 days, the 0.5% dose produced the most consistent improvement in body weight gain and feed efficiency without increasing feed intake. Supplementation also improved several blood indicators related to antioxidant defense and antibody-mediated immunity, while routine blood biochemical indicators were unchanged. The 0.5% dose changed the composition, but not the overall diversity, of the intestinal microbiota and increased several bacteria associated with intestinal health. It also altered serum metabolites involved mainly in amino acid, lipid, and energy metabolism. Higher doses did not provide further benefits. These results suggest that including 0.5% of this plant complex in the diet may support the growth and physiological regulation of yellow-feathered broilers; however, larger studies under different production conditions are needed to confirm its practical value.

## 1. Introduction

The rapid development of intensive poultry production has substantially increased the demand for efficient and sustainable strategies to improve animal health and production efficiency. Antibiotic growth promoters have historically been incorporated into poultry diets to promote growth, improve feed utilization, and reduce the incidence of infectious diseases [1]. However, the extensive or inappropriate use of antimicrobial agents in food-producing animals can promote the selection and dissemination of antimicrobial-resistant bacteria and resistance genes, with potential consequences for both animal and human health [2]. Moreover, failure to comply with approved dosages and withdrawal periods may result in antibiotic residues in poultry products, raising additional food-safety concerns [3]. In response to these risks, the use of medically important antimicrobials for growth-promotion purposes has been restricted or prohibited in many countries and regions [1,4]. Therefore, the development of safe, effective, and environmentally sustainable alternatives to antibiotic growth promoters has become an important research priority in modern livestock and poultry production.

Plant-derived functional feed additives have received increasing attention as promising alternatives to antibiotic growth promoters because of their natural origin, diverse bioactive constituents, and multiple biological activities. These natural products contain various functional components, including polysaccharides, flavonoids, saponins, and phenolic compounds, which may exert antioxidant, immunomodulatory, anti-inflammatory, and intestinal microbiota-regulating effects [5]. The antioxidant properties of these compounds are particularly relevant to poultry productivity because modern broilers are frequently exposed to oxidative challenges arising from rapid growth, high metabolic activity, environmental stress, immune stimulation, and intensive production conditions [6]. When the generation of reactive oxygen and nitrogen species exceeds the capacity of endogenous antioxidant defenses, oxidative stress can damage cellular lipids, proteins, and DNA and interfere with normal cellular signaling and physiological homeostasis [6]. The gastrointestinal tract is particularly susceptible to oxidative injury because it is continuously exposed to dietary pro-oxidants, microbial metabolites, inflammatory stimuli, and environmental stressors. Excessive oxidative stress can impair epithelial integrity, intestinal immune-barrier function, digestive and absorptive capacity, and microbial homeostasis [7]. Experimental evidence further indicates that production-related stress can increase intestinal permeability and adversely affect physiological and production-associated traits in broilers [8]. Consequently, oxidative damage may decrease nutrient utilization and divert nutrients and metabolic energy away from tissue growth toward stress adaptation, immune activation, and cellular repair, ultimately compromising body-weight gain and feed efficiency. Therefore, maintaining an appropriate oxidative balance may help preserve intestinal and systemic physiological functions and support broiler health and productive performance. Herbal complexes derived from medicinal and edible plants may exert complementary or potentially synergistic biological effects through multiple bioactive constituents and regulatory targets, thereby supporting antioxidant defense, intestinal health, immune competence, and production performance [9].

*Astragalus membranaceus*, a traditional medicinal and edible plant, is widely used in animal nutrition because of its abundant bioactive compounds, including polysaccharides, saponins, and flavonoids [10,11,12]. Previous studies have demonstrated that *Astragalus membranaceus* and its bioactive derivatives can enhance antioxidant defenses and immune responses, modulate intestinal microbial communities, and improve digestive function and feed utilization efficiency in poultry [10,11,13,14]. In particular, Astragalus polysaccharides have been reported to promote intestinal immune homeostasis and barrier health by modulating host immune responses, inflammatory signaling, and gut microbial communities in poultry [9,15,16].

*Eucommia ulmoides* leaf represent another valuable botanical resource with considerable potential for application in animal production. They contain diverse bioactive constituents, including chlorogenic acid, flavonoids, and polysaccharides, which are associated with antioxidant, anti-inflammatory, lipid metabolism-regulating, and intestinal protective activities [17,18]. Studies in poultry have demonstrated that dietary supplementation with *Eucommia ulmoides* leaf extract can improve growth or production performance, enhance antioxidant defense and immune status, regulate lipid metabolism, and alter serum metabolic profiles and intestinal microbial communities [19,20]. Furthermore, chlorogenic acid, a major phenolic constituent of *E. ulmoides* leaf, has been shown to improve intestinal morphology and barrier integrity, alleviate inflammatory responses, and modulate gut microbial communities in broilers exposed to environmental or immune stress [21,22]. Collectively, these findings highlight the potential value of *E. ulmoides* leaves as a natural functional feed additive for poultry.

Despite this biological rationale, it remains unclear whether the *Eucommia ulmoides* leaf–*Astragalus membranaceus* complex can influence broiler growth through coordinated regulation of intestinal microbiota and host metabolism. Most available poultry studies have investigated the effects of *A. membranaceus*-derived polysaccharides or *E. ulmoides* leaf extracts separately, particularly in relation to growth performance, antioxidant capacity, immune regulation, and intestinal health [23,24]. Consequently, evidence regarding the complementary or interactive effects of these two botanical resources remains limited. Yellow-feathered chickens include numerous Chinese indigenous breeds and constitute an important component of poultry production in China. Compared with fast-growing commercial broilers, they generally have a longer production cycle but are highly valued for their distinctive carcass characteristics, meat quality, flavor-related metabolic profiles, and consumer preference [25,26]. Their annual sales reach approximately four billion birds in China, further demonstrating their considerable industrial and economic importance [27]. Therefore, improving growth and feed utilization while maintaining the physiological and intestinal health of yellow-feathered broilers has substantial practical significance. Nevertheless, previous investigations of botanical additives have frequently focused on individual ingredients or isolated physiological endpoints [23,24]. Although recent poultry studies have demonstrated the value of linking microbial alterations with intestinal and systemic metabolic responses [28,29], few studies have integrated bioactive-component characterization and in vitro activity assessment with growth performance, serum physiological indices, intestinal microbiota, and host metabolomic profiles. This fragmented evidence limits a comprehensive understanding of whether intestinal microbial changes are associated with systemic metabolic responses and production performance, highlighting the need for an integrated evaluation of this herbal complex in yellow-feathered broilers.

## 2. Materials and Methods

### 2.1. Preparation and Characterization of the Eucommia ulmoides Leaf-Astragalus membranaceus Complex

The dried of the *Eucommia ulmoides* leaf-*Astragalus membranaceus* complex were obtained from Huanghe Chinese Medicinal Materials Market (Lanzhou, China). The botanical materials were authenticated according to the morphological characteristics and standards described in the Chinese Pharmacopoeia. After removing impurities, the herbal materials were dried, ground into powder, passed through an 80-mesh sieve, and stored in sealed containers under dry conditions until further use. The *Eucommia ulmoides* leaf-*Astragalus membranaceus* complex was prepared by mixing *Astragalus membranaceus* root powder and *Eucommia ulmoides* leaf powder at a ratio of 1:1 (*w*/*w*). The mixed powder was used for subsequent chemical composition analysis, antioxidant activity evaluation, and animal feeding experiments. For preparation of the aqueous extract, the herbal powder was extracted with distilled water at a solid-to-liquid ratio of 1:10 (*w*/*v*) and soaked for 30 min. The mixture was decocted for 60 min and filtered through eight layers of gauze. The residue was subsequently extracted with distilled water at a ratio of 1:8 (*w*/*v*) for another 60 min. The filtrates from both extractions were combined, centrifuged at 4500 rpm for 5 min, and concentrated under reduced pressure at 55 °C. The concentrated extract was pre-frozen at −80 °C for 12 h and freeze-dried for 24–48 h to obtain the dried herbal extract powder, which was stored at 4 °C until analysis.

### 2.2. Determination of Active Components

The major bioactive compounds in the herbal complex were determined using high-performance liquid chromatography (HPLC) and colorimetric assays. The contents of chlorogenic acid and calycosin-7-O-β-D-glucoside were determined using an Agilent 1260 HPLC system equipped (Agilent Technologies, Santa Clara, CA, USA) with a Zorbax Bonus-RP column (Agilent Technologies, Santa Clara, CA, USA, 4.6 mm × 250 mm, 5 μm). The mobile phase consisted of acetonitrile and 0.4% phosphoric acid aqueous solution with gradient elution. The column temperature was maintained at 30 °C, the flow rate was set at 1.0 mL/min, the detection wavelength was 290 nm, and the injection volume was 20 μL. Calibration curves were established using corresponding reference standards. The total polysaccharide content was determined using the phenol–sulfuric acid method with glucose as the standard. Total flavonoid content was measured using the sodium nitrite–aluminum nitrate colorimetric method with rutin as the standard, and total saponin content was determined using the vanillin–sulfuric acid method with ginsenoside Rb1 as the reference standard.

### 2.3. Determination of Antioxidant Activity

The antioxidant capacity of the herbal complex was evaluated using DPPH, ABTS, hydroxyl radical (•OH) scavenging assays, and ferric reducing antioxidant power (FRAP) analysis. Different concentrations of herbal extract solutions were incubated with corresponding radical reaction systems, and absorbance values were measured at 517 nm (DPPH), 734 nm (ABTS), and 520 nm (•OH). Vitamin C was used as the positive control. The scavenging capacity was expressed as inhibition percentage and IC50 values. The reducing capacity was evaluated using the FRAP method, and the antioxidant activity was expressed as FeSO_4_ equivalent reducing capacity.

### 2.4. Experimental Animals and Dietary Treatments

A total of 200 healthy one-day-old male yellow-feathered broilers were randomly allocated to four dietary treatments, with five replicate cages per treatment and 10 birds per cage. The birds were housed in three-tier cages for 42 days with ad libitum access to feed and water; stocking density remained constant, with no transfer or regrouping. The treatments included a control group (CG) fed a basal diet, and three experimental groups fed the basal diet supplemented with 0.5%, 1.0%, and 2.0% *Eucommia ulmoides* leaf–*Astragalus membranaceus* complex, designated EG (0.5%), EG (1.0%), and EG (2.0%), respectively. The basal diets, supplied by Shanxi Aoyoute Feed Co., Ltd. (Yuncheng, China), comprised a starter diet for days 1–21 (diet 881) and a grower–finisher diet for days 22–42 (diet 882), with their composition and nutrient levels shown in Table 1. The temperature in the poultry house was maintained at 34–35 °C from days 1 to 3 and at 32–34 °C from days 4 to 7. Thereafter, it was reduced by 2–3 °C per week and maintained at 20–22 °C from days 36 to 42. A photoperiod of 24 h light:0 h darkness was provided from days 1 to 3. Beginning on day 4, the daily light period was gradually shortened by 0.5 h per day until a photoperiod of 16 h light:8 h darkness was reached on day 19; this schedule was maintained through day 42, with the dark period occurring at night. Light intensity was maintained at 20 lx from days 1 to 7, 10 lx from days 8 to 21, and 8 lx from days 22 to 42.

### 2.5. Sample Collection

On days 21 and 42, seven birds from each group were randomly selected for sample collection. Before morning feeding, the selected birds were anesthetized with sodium pentobarbital (50 mg/kg body weight) administered intravenously via the wing vein until loss of consciousness. Subsequently, blood samples were collected from the wing vein and left to stand at room temperature. Serum was separated by centrifugation at 3000 rpm for 15 min and stored at −80 °C until subsequent analyses of serum biochemical, antioxidant, and immune-related parameters. Following blood collection, the birds were humanely euthanized by cervical dislocation while under deep anesthesia. Immediately after euthanasia, jejunal and cecal contents were collected aseptically, snap-frozen in liquid nitrogen, and stored at −80 °C until microbiota analysis.

### 2.6. Determination of Serum Antioxidant, Immune and Biochemical Parameters

Serum antioxidant indices, including superoxide dismutase (SOD) and glutathione peroxidase (GSH-Px), were measured using commercial assay kits according to the manufacturer’s instructions. Serum immunoglobulin levels, including IgA, IgM, and IgG, were determined using enzyme-linked immunosorbent assay (ELISA). Serum biochemical parameters, including total protein (TP), albumin (ALB), globulin (GLOB), uric acid (UA), glucose (GLU), calcium (Ca^2+^), phosphorus (P), sodium (Na^+^), potassium (K^+^), and chloride (Cl^−^), were analyzed using an automatic biochemical analyzer.

### 2.7. Gut Microbiota Sequencing and Bioinformatic Analysis

Genomic DNA was extracted from cecal and jejunal contents using a commercial DNA extraction kit. DNA concentration and purity were evaluated using a NanoDrop spectrophotometer. The V3–V4 region of bacterial 16S rRNA genes was amplified using universal primers. PCR products were purified using AMPure XP magnetic beads (Beckman Coulter, Brea, CA, USA), quantified using an Agilent 2100 Bioanalyzer (Agilent Technologies, Santa Clara, CA, USA), and sequenced on an Illumina MiSeq platform. Raw sequencing reads were processed using the QIIME2 pipeline (Illumina, San Diego, CA, USA). After quality filtering, trimming, and chimera removal, high-quality sequences were denoised using the DADA2 algorithm to generate amplicon sequence variants (ASVs). Taxonomic classification was performed using the SILVA database. Alpha diversity indices (Chao1, Shannon, Simpson, and ACE), beta diversity analysis based on Bray–Curtis distance, principal coordinate analysis (PCoA), differential abundance analysis, and microbial correlation analysis were performed to evaluate microbial community alterations.

### 2.8. Serum Metabolomics Analysis

Serum metabolites were extracted by methanol precipitation. Briefly, samples were vortexed, sonicated, centrifuged, and filtered through 0.22 μm membranes. Metabolomic profiling was performed using ultra-performance liquid chromatography coupled with tandem mass spectrometry (UPLC–MS/MS). Quality-control samples were prepared by pooling equal aliquots from all samples and were injected periodically throughout the analytical sequence to monitor instrument stability and analytical reproducibility. Raw data were processed for peak extraction, alignment, normalization, and metabolite identification. Multivariate analyses, including principal component analysis (PCA) and orthogonal partial least-squares discriminant analysis (OPLS–DA), were subsequently performed. For univariate analysis, the resulting *p* values were adjusted using the Benjamini–Hochberg procedure to control the false discovery rate (FDR) associated with multiple testing. Metabolites with a variable importance in projection (VIP) > 1, a nominal *p* < 0.05, and an FDR-adjusted *p* < 0.05 were considered differentially abundant. Differential metabolites were annotated using the Kyoto Encyclopedia of Genes and Genomes (KEGG) database, and the *p* values obtained from pathway-enrichment analysis were likewise adjusted using the Benjamini–Hochberg procedure, with *p* < 0.05 considered statistically significant.

### 2.9. Statistical Analysis

Growth performance data are presented as the mean ± standard error (mean ± SE). All other data were statistically analyzed and graphically presented using GraphPad Prism version 9.5 (GraphPad Software, Boston, MA, USA). Before parametric analysis, the normality and homogeneity of variance of the data were assessed. Data that satisfied the assumptions of normality and homogeneity of variance were analyzed using one-way analysis of variance (ANOVA). When the overall treatment effect was significant, Tukey’s multiple-comparisons test was subsequently performed for pairwise comparisons between groups. Differences were considered statistically significant at *p* < 0.05.

## 3. Results

### 3.1. Determination Results of Nutritional Components in Eucommia ulmoides Leaf–Astragalus membranaceus Complex

The *Eucommia ulmoides* leaf-*Astragalus membranaceus* complex is rich in nutrients. It contains 94.00% dry matter and 21.40% crude protein (dry matter basis), with 16.45% being effective crude protein. Soluble protein makes up 29.00% of the total protein, while rumen-undegradable and rumen-degradable proteins account for 31.00% and 69.00%, respectively. The fiber components include 36.80% acid detergent fiber, 34.00% neutral detergent fiber, and 2.30% lignin. Non-fiber carbohydrates, starch, fat, and ash are present at 29.74%, 8.46%, 2.41%, and 8.93%, respectively. The complex’s total digestible nutrients are 69.00%, and it contains minerals such as calcium, phosphorus, magnesium, potassium, sulfur, and chlorine. The calcium-to-phosphorus ratio is 2.76, the nitrogen-to-sulfur ratio is 11.81, and the potassium-to-magnesium ratio is 5.69. These findings suggest that the *Eucommia ulmoides* leaf-*Astragalus membranaceus* complex offers active ingredients and essential nutrients, providing nutritional support for broilers (see Table 2 for specific results).

### 3.2. Determination of the Content of Active Components in the Eucommia ulmoides Leaf-Astragalus membranaceus Complex

**Figure 1:**
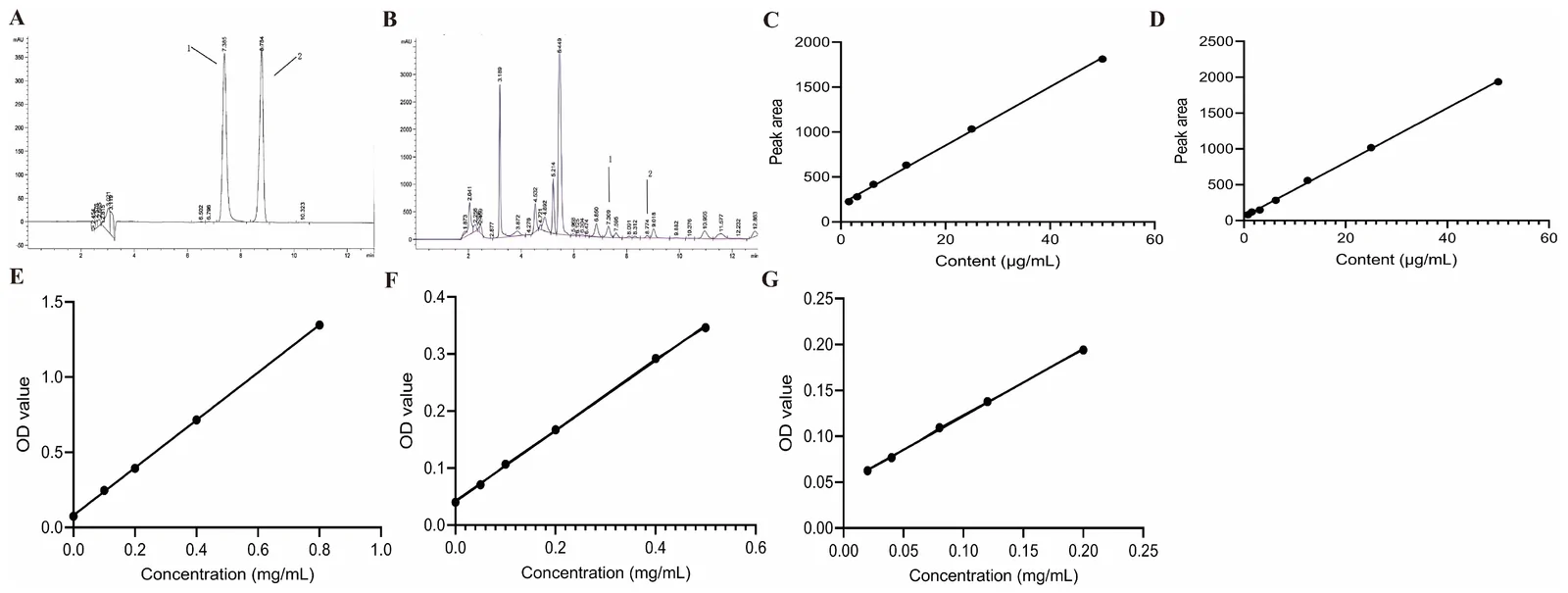
Content of active components in *Eucommia ulmoides* leaf-*Astragalus membranaceus* complex. Note: (**A**): Chromatogram of chlorogenic acid and calycosin-7-O-β-D-glucoside standard samples; (**B**): Chromatogram of chlorogenic acid and calycosin-7-O-β-D-glucoside in the *Eucommia ulmoides* leaf-*Astragalus membranaceus* complex; (**C**): Standard curve of chlorogenic acid; (**D**): Standard curve of calycosin-7-O-β-D-glucoside; (**E**): Standard curve of total polysaccharides in the *Eucommia ulmoides* leaf-*Astragalus membranaceus* complex; (**F**): Standard curve of total flavonoids in the *Eucommia ulmoides* leaf-*Astragalus membranaceus* complex; (**G**): Standard curve of total saponins in the *Eucommia ulmoides* leaf-*Astragalus membranaceus* complex.

HPLC analysis revealed that chlorogenic acid and calycosin-7-O-β-D-glucoside were effectively separated, with retention times of 7.309 min and 8.774 min, respectively (Figure 1A,B). The linear ranges for chlorogenic acid and calycosin-7-O-β-D-glucoside were 0.8374–49.4898 μg/mL and 0.6721–49.6630 μg/mL, with correlation coefficients R^2^ of 0.9986 and 0.9992, respectively (Figure 1C,D). Precision, repeatability, stability, and recovery tests all satisfied methodological standards. Parallel determinations showed average contents of 475.05 μg/g for chlorogenic acid and 422.84 μg/g for calycosin-7-O-β-D-glucoside in the complex. Colorimetric assays indicated total polysaccharides, flavonoids, and saponins contents were 46.53%, 1.0034%, and 0.3126%, respectively (Figure 1E–G). These results suggest that the *Eucommia ulmoides* leaf-*Astragalus membranaceus* complex is rich in polysaccharides and contains minor amounts of flavonoids and saponins.

### 3.3. Determination of the Antioxidant Activity of the Eucommia ulmoides Leaf-Astragalus membranaceus Complex

**Figure 2:**
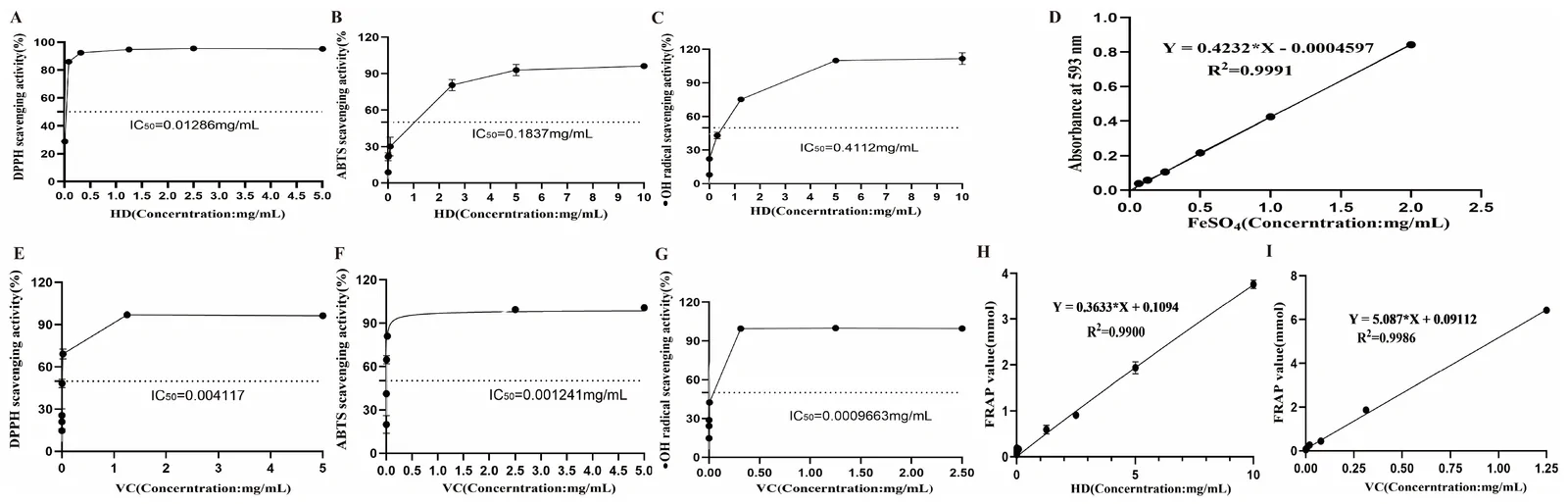
Antioxidant activity of *Eucommia ulmoides* leaf-*Astragalus membranaceus* complex. Note: (**A**): DPPH radical scavenging ability in the *Eucommia ulmoides* leaf-*Astragalus membranaceus* complex; (**B**): ABTS radical scavenging ability in the *Eucommia ulmoides* leaf-*Astragalus membranaceus* complex; (**C**): •OH radical scavenging ability in the *Eucommia ulmoides* leaf-*Astragalus membranaceus* complex; (**D**): Standard curve of Fe^3+^ reducing ability; (**E**): DPPH radical scavenging ability of VC; (**F**): ABTS radical scavenging ability of VC; (**G**): •OH radical scavenging ability of VC; (**H**): Fe^3+^ reducing ability of the *Eucommia ulmoides* leaf-*Astragalus membranaceus* complex; (**I**): Fe^3+^ reducing ability of VC.

The *Eucommia ulmoides* leaf-*Astragalus membranaceus* complex has the ability to scavenge DPPH, ABTS, and •OH free radicals, and the scavenging rate increases with the increase in concentration, showing an obvious dose-effect relationship (Figure 2A–I). The IC_50_ values of the complex for DPPH, ABTS, and •OH free radicals are 0.01286 mg/mL, 0.1837 mg/mL, and 0.4112 mg/mL, respectively; the IC_50_ values of the positive control VC are 0.004117 mg/mL, 0.001241 mg/mL, and 0.0009663 mg/mL, respectively. The FRAP results show that the Fe^3+^ reducing ability of the complex is 0.1094 mmol FeSO_4_/g, which is lower than that of VC, but it still shows a certain reducing ability. The results indicate that the *Eucommia ulmoides* leaf–*Astragalus membranaceus* complex has good antioxidant activity.

### 3.4. Effects of Dietary Eucommia ulmoides Leaf-Astragalus membranaceus Complex on Growth Performance of Yellow-Feathered Broilers

Dietary supplementation with the *Eucommia ulmoides* leaf–*Astragalus membranaceus* complex improved the growth performance of yellow-feathered broilers, with the most pronounced effects observed in the EG (0.5%) group (Table 3). At 1 day of age, no significant differences were observed in initial BW among CG, EG (0.5%), EG (1%), and EG (2%) groups, indicating that the broilers had comparable initial body weights. During the starter phase, EG supplementation significantly increased BW at 21 d and improved ADG from 1 to 21 d. Compared with CG, the EG (0.5%) group showed significantly higher BW at 21 d and ADG from 1 to 21 d (*p* < 0.05), whereas EG (1%) and EG (2%) showed intermediate values. In addition, FCR from 1 to 21 d was significantly reduced in the EG (0.5%) and EG (2%) groups compared with CG (*p* < 0.05), while FI was not significantly affected among treatments (*p* > 0.05). During the grower phase, EG supplementation continued to improve growth performance. At 42 d, BW was significantly higher in the EG (0.5%) group than in CG, EG (1%), and EG (2%) groups (*p* < 0.05). Similarly, ADG from 21 to 42 d was significantly increased in the EG (0.5%) group compared with CG and EG (2%) groups (*p* < 0.05), while EG (1%) showed an intermediate response. FCR from 21 to 42 d was significantly lower in the EG (0.5%) group than in CG (*p* < 0.05), indicating improved feed utilization efficiency. However, FI during this period showed no significant difference among groups (*p* > 0.05). Overall, dietary EG supplementation improved growth performance mainly by enhancing feed conversion efficiency rather than increasing feed intake. Among the tested supplementation levels, EG (0.5%) showed the most consistent beneficial effects on BW, ADG, and FCR throughout the 42-day feeding period, suggesting that 0.5% EG may be an optimal dietary inclusion level for yellow-feathered broilers.

### 3.5. Effects of Eucommia ulmoides Leaf-Astragalus membranaceus Complex on Serum Antioxidant and Immune Indices of Yellow-Feather Broiler

**Figure 3:**
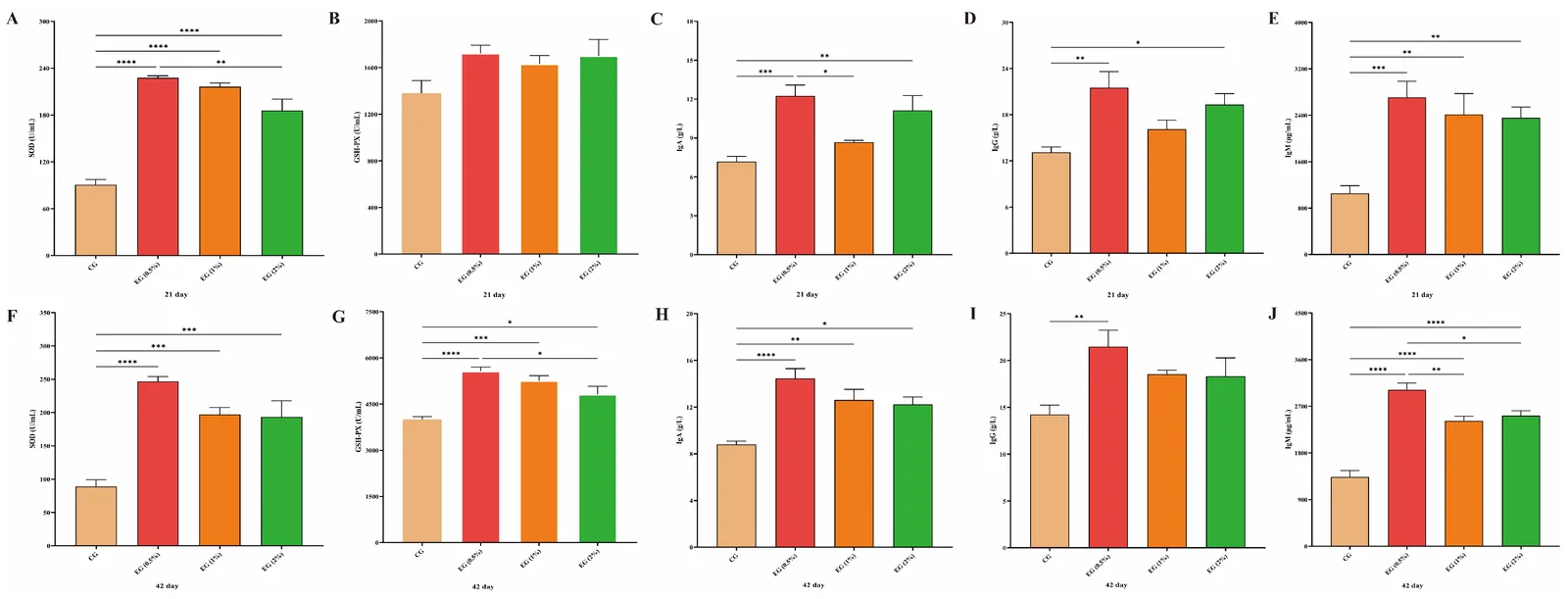
Impact of *Eucommia ulmoides* leaf-*Astragalus membranaceus* complex on serum antioxidant and immune markers in yellow-feather broilers. Note: (**A**): SOD content at 21 day; (**B**): GSH-Px content at 21 day; (**C**): IgA content at 21 day; (**D**): IgG content at 21 day; (**E**): IgM content at 21 days; (**F**): SOD content at 42 day; (**G**): GSH-Px content at 42 day; (**H**): IgA content at 42 day; (**I**): IgG content at 42 day; (**J**): IgM content at 42 day. * *p* < 0.05, ** *p* < 0.01, *** *p* < 0.001 and **** *p* < 0.0001.

Dietary supplementation with the *Eucommia ulmoides* leaf–*Astragalus membranaceus* complex significantly affected serum antioxidant enzyme activities and immunoglobulin concentrations in yellow-feathered broilers (Figure 3A–J). At 21 days of age, all EG-supplemented groups exhibited significantly higher serum SOD activity than the control group (*p* < 0.0001), whereas GSH-Px activity remained unaffected (*p* > 0.05). Serum IgA and IgG concentrations were significantly increased in the EG (0.5%) and EG (2%) groups compared with CG (*p* < 0.05), while serum IgM concentration was significantly elevated in all EG-supplemented groups (*p* < 0.01). At 42 days of age, serum SOD and GSH-Px activities, as well as IgA and IgM concentrations, were significantly higher in all EG-supplemented groups than in CG (*p* < 0.05). However, a significant increase in serum IgG concentration was observed only in the EG (0.5%) group (*p* < 0.01). Among the supplementation treatments, the EG (0.5%) group generally exhibited the highest antioxidant enzyme activities and immunoglobulin concentrations, whereas increasing the inclusion level to 1% and 2% produced no additional improvement. Overall, these results indicate that dietary EG supplementation enhanced serum antioxidant status and humoral immune-related indices, with the most consistent responses observed at the 0.5% inclusion level.

### 3.6. Effects of Eucommia ulmoides Leaf-Astragalus membranaceus Complex on Serum Biochemical Indices of Yellow-Feathered Broilers

**Figure 4:**
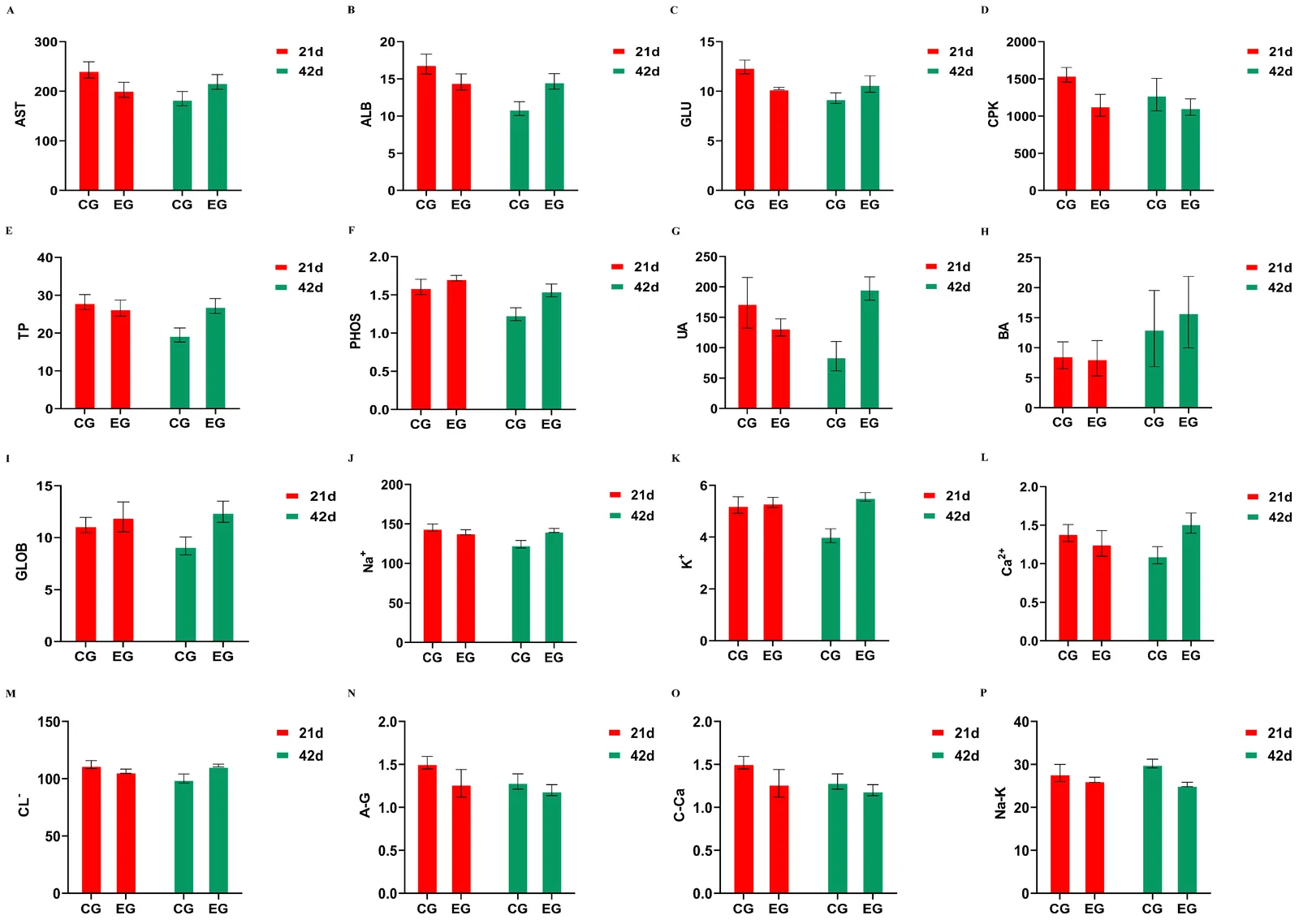
Impact of *Eucommia ulmoides* leaf-*Astragalus membranaceus* complex on serum biochemical markers in yellow-feather broilers. Note: (**A**): AST levels at 21 and 42 days; (**B**): ALB levels at 21 and 42 days; (**C**): GLU levels at 21 and 42 days; (**D**): CPK levels at 21 and 42 days; (**E**): TP levels at 21 and 42 days; (**F**): PHOS levels at 21 and 42 days; (**G**): UA levels at 21 and 42 days; (**H**): BA levels at 21 and 42 days; (**I**): GLOB levels at 21 and 42 days; (**J**): Na levels at 21 and 42 days; (**K**): K levels at 21 and 42 days; (**L**): Ca levels at 21 and 42 days; (**M**): CL levels at 21 and 42 days; (**N**): A-G levels at 21 and 42 days; (**O**): C-Ca levels at 21 and 42 days; (**P**): Na-K levels at 21 and 42 days.

Dietary supplementation with *the Eucommia ulmoides* leaf–*Astragalus membranaceus* complex had no significant effect on the serum biochemical parameters of yellow-feathered broilers (Figure 4A–P). At 21 days of age, no significant differences were observed between the EG-supplemented groups and the control group in serum AST, ALB, GLU, CPK, TP, PHOS, UA, BA, GLOB, Na^+^, K^+^, Ca^2+^, Cl^−^, A/G, C-Ca, or the Na^+^/K^+^ ratio (*p* > 0.05). Similarly, at 42 days of age, none of these serum biochemical parameters differed significantly between the EG-supplemented groups and CG (*p* > 0.05). Overall, under the present experimental conditions, dietary EG supplementation did not significantly affect serum biochemical parameters related to protein metabolism, hepatic and muscular function, or mineral and electrolyte homeostasis, and these parameters remained relatively stable at both sampling time points. These findings indicate that EG did not induce detectable serum biochemical abnormalities at the inclusion levels and during the feeding period evaluated, providing preliminary evidence supporting its safety in yellow-feathered broilers.

### 3.7. Effects of Eucommia ulmoides Leaf–Astragalus membranaceus Complex on the Intestinal Flora of Yellow-Feather Broilers

#### 3.7.1. Effects of *Eucommia ulmoides* Leaf–*Astragalus membranaceus* Complex on the Jejunal Flora of Yellow-Feather Broilers

**Figure 5:**
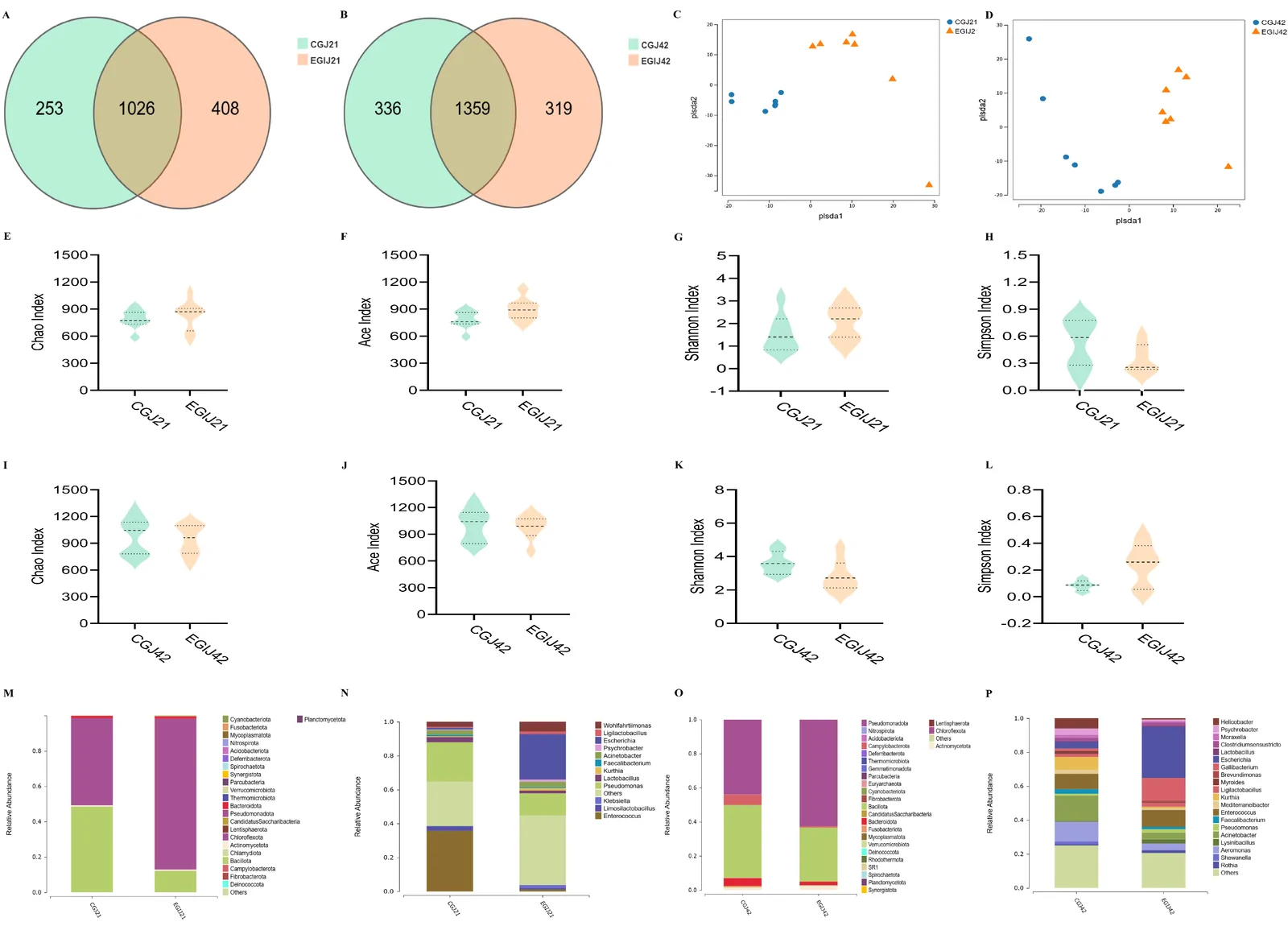
Effects of *Eucommia ulmoides* leaf–*Astragalus membranaceus* complex supplementation on the structure and diversity of the jejunal microbiota in yellow-feathered broilers. Note: (**A**,**B**) Venn diagrams showing the shared and unique operational taxonomic units (OTUs) of the jejunal microbiota in the CGJ and EGIJ groups at 21 and 42 days of age, respectively; (**C**,**D**) partial least squares discriminant analysis (PLS-DA) score plots of the jejunal microbial communities at 21 and 42 days of age, respectively; (**E**–**L**) box plots of the alpha diversity indices (Chao1, ACE, Shannon, and Simpson) of the jejunal microbiota in the CGJ and EGIJ groups at 21 and 42 days of age; (**M**,**N**) relative abundances of the dominant bacterial phyla in the jejunal contents at 21 and 42 days of age, respectively; and (**O**,**P**) relative abundances of the dominant bacterial genera in the jejunal contents at 21 and 42 days of age, respectively. CGJ, jejunal samples from the control group; EGIJ, jejunal samples from the *Eucommia ulmoides* leaf–*Astragalus membranaceus* complex-supplemented group.

Venn analysis revealed differences in OTU profiles between the EG and CG groups at both 21 and 42 days of age (Figure 5A,B). PLS-DA analysis demonstrated distinct separation of microbial communities between EG and CG groups, indicating that EG supplementation altered the overall structure of the jejunal microbiota (Figure 5C,D). However, no significant differences were observed in alpha diversity indices between the two groups (Figure 5E–L). Taxonomic analysis revealed that EG supplementation modified the relative abundance of dominant bacterial taxa. At 21 days of age, EG supplementation increased the relative abundances of *Pseudomonadota* and *Cyanobacteriota* while decreasing those of *Bacillota* and *Candidatus Saccharibacteria*. At the genus level, EG supplementation increased the relative abundance of Escherichia and decreased the abundances of *Enterococcus* and *Pseudomonas* (Figure 5M,N). At 42 days of age, EG supplementation increased the relative abundance of *Pseudomonadota* and *Deinococcota* and decreased that of *Bacillota* and *Campylobacterota*. Corresponding changes were also observed at the genus level, including alterations in the abundances of *Escherichia*, *Gallibacterium*, *Acinetobacter*, and *Helicobacter* (Figure 5O,P). These findings indicate that EG supplementation reshaped the jejunal microbial community composition without markedly affecting overall microbial diversity.

#### 3.7.2. Effects of *Eucommia ulmoides* Leaf-*Astragalus membranaceus* Complex Supplementation on Cecal Microbiota Composition of Yellow-Feathered Broilers

**Figure 6:**
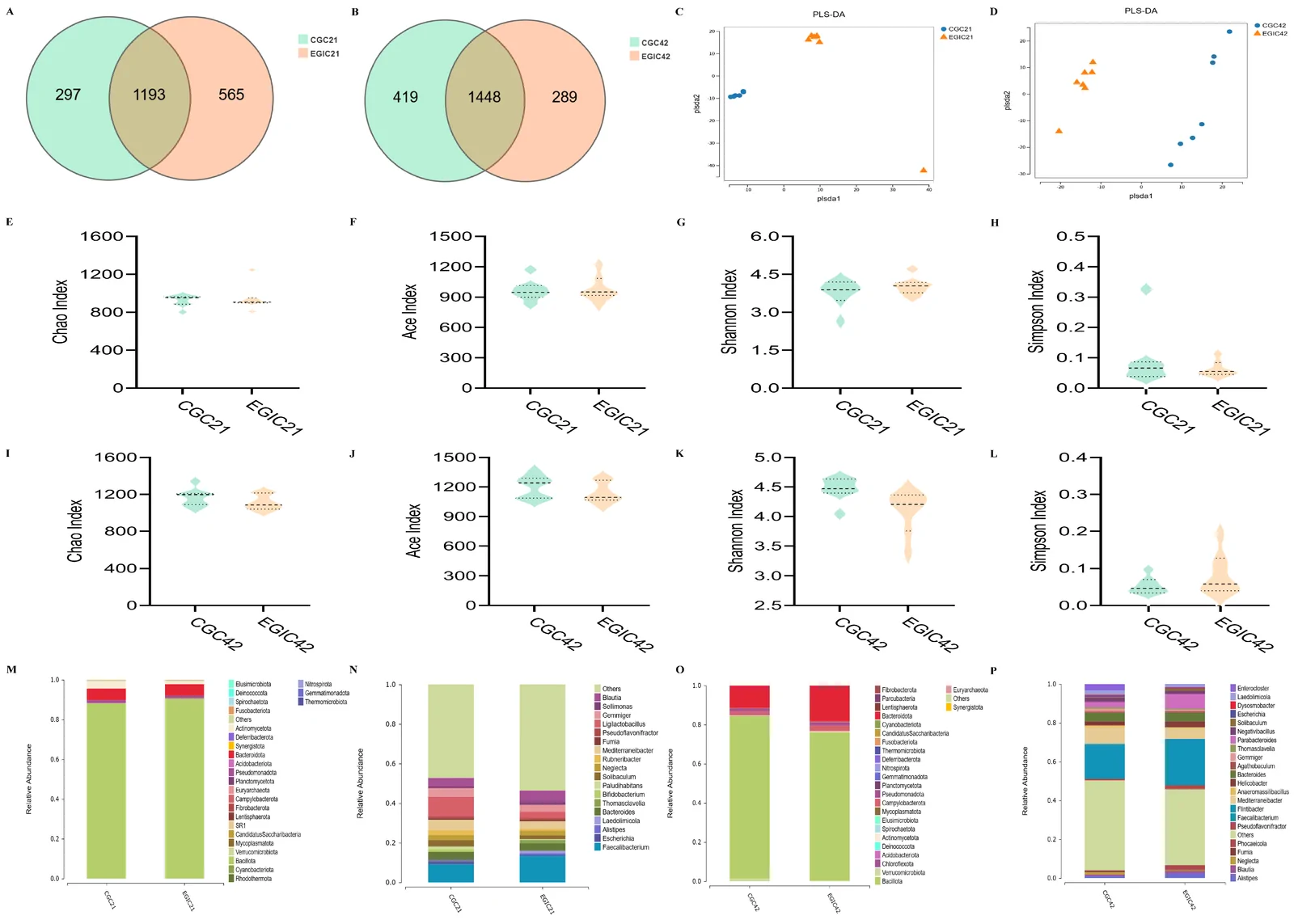
Effects of *Eucommia ulmoides* leaf–*Astragalus membranaceus* complex supplementation on the structure and diversity of the cecal microbiota in yellow-feathered broilers. Note: (**A**,**B**) Venn diagrams showing the shared and unique operational taxonomic units (OTUs) of the cecal microbiota in the CGC and EGIC groups at 21 and 42 days of age, respectively; (**C**,**D**) partial least squares discriminant analysis (PLS-DA) score plots of the cecal microbial communities at 21 and 42 days of age, respectively; (**E**–**L**) box plots of the alpha diversity indices (Chao1, ACE, Shannon, and Simpson) of the cecal microbiota in the CGC and EGIC groups at 21 and 42 days of age; (**M**,**N**) relative abundances of the dominant bacterial phyla in the cecal contents at 21 and 42 days of age, respectively; and (**O**,**P**) relative abundances of the dominant bacterial genera in the cecal contents at 21 and 42 days of age, respectively. CGC, cecal samples from the control group; EGIC, cecal samples from the *Eucommia ulmoides* leaf–*Astragalus membranaceus* complex-supplemented group.

Dietary supplementation with the *Eucommia ulmoides* leaf–*Astragalus membranaceus* complex significantly modulated cecal microbiota composition and community structure in yellow-feathered broilers. Venn analysis showed that EG supplementation altered the OTU composition of the cecal microbiota at both 21 and 42 days, with a greater number of unique microbial features observed in the EG group compared with CG group (Figure 6A,B). PLS-DA analysis revealed clear separation between CG and EG groups at both sampling time points, indicating that EG supplementation induced distinct changes in cecal microbial community structure (Figure 6C,D). However, alpha diversity analysis, including Chao1, Ace, Shannon, and Simpson indices, showed no significant differences between groups, suggesting that EG primarily influenced microbial composition rather than overall diversity (Figure 6E–L). At the phylum level, EG supplementation altered the relative abundance of dominant bacterial taxa, including *Bacillota* and *Bacteroidota*. At 21 days, EG supplementation increased the abundance of *Fusobacteriota*, *Campylobacterota*, *Spirochaetota*, *Synergistota*, and *Candidatus Saccharibacteria*, while decreasing *Actinomycetota* and *Verrucomicrobiota*. At the genus level, EG promoted the enrichment of beneficial bacteria, including *Faecalibacterium* and *Blautia*, and modulated the abundance of several other microbial genera (Figure 6M,N). After 42 days of feeding, EG further influenced microbial composition by increasing the abundance of health-associated genera, including *Faecalibacterium* and *Parabacteroides*, while reducing some potentially unfavorable bacterial taxa (Figure 6O,P). Collectively, these results demonstrate that dietary EG supplementation reshaped cecal microbial community structure and selectively regulated bacterial composition without markedly affecting microbial diversity, suggesting that EG may improve intestinal ecological balance and contribute to enhanced nutrient metabolism and physiological health in yellow-feathered broilers.

### 3.8. Effects of Eucommia ulmoides Leaf–Astragalus membranaceus Complex on the Serum Metabolome of Yellow-Feather Broilers

**Figure 7:**
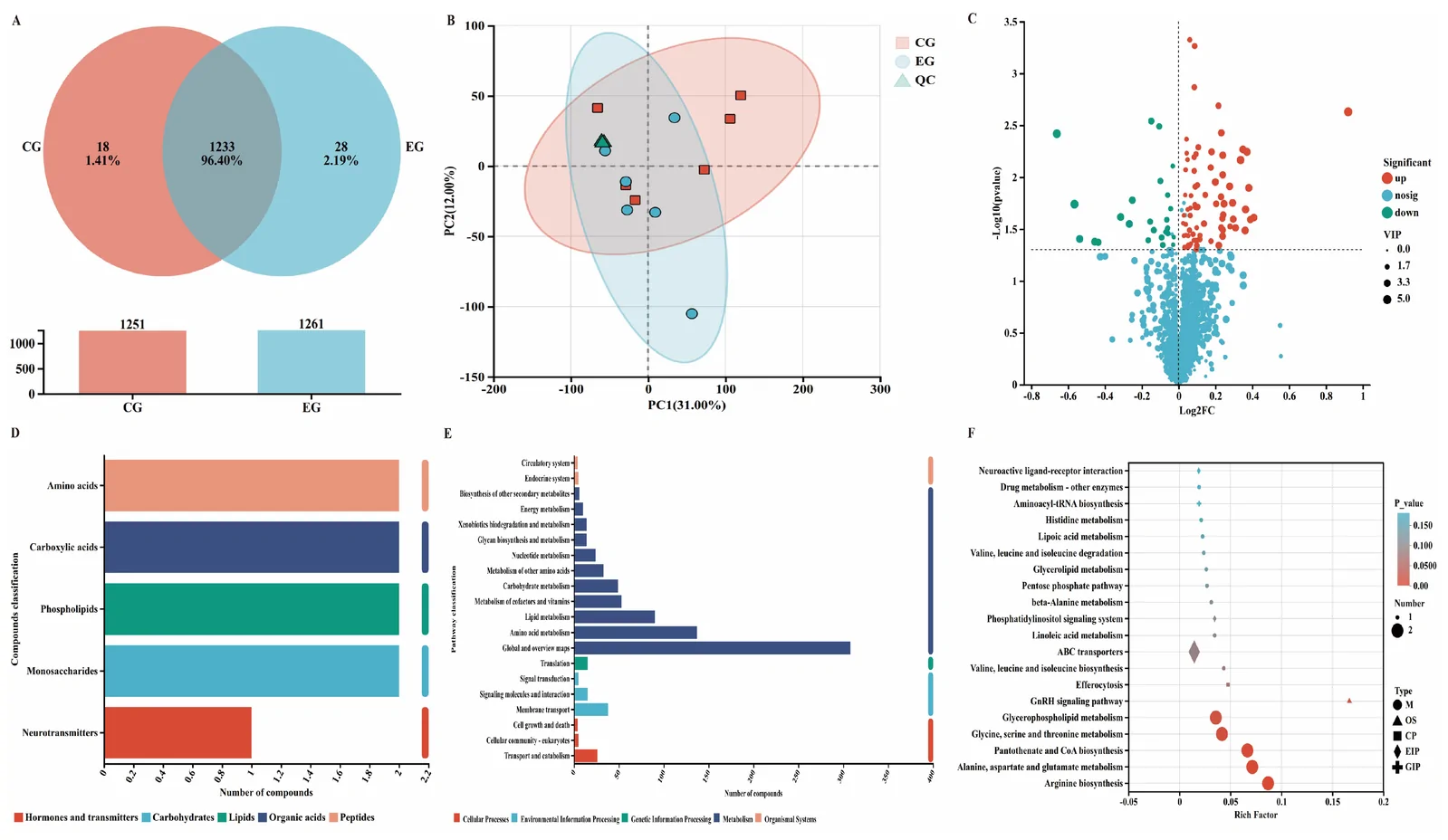
Effects of *Eucommia ulmoides* leaf–*Astragalus membranaceus* complex on the serum metabolome of yellow-feather broilers. Note: (**A**): Venn diagram of serum metabolites and statistics of the number of unique metabolites in the control group (CG) and the *Eucommia ulmoides* leaf–*Astragalus membranaceus* complex group (EG); (**B**): Scatter plot of the distribution of serum metabolites in the CG, EG, and quality control group (QC) based on principal component analysis (PCA); (**C**): Volcano plot of orthogonal partial least squares discriminant analysis (OPLS—DA), showing the differential metabolites between the CG and EG groups (red for up—regulated and green for down—regulated); (**D**): Main classification statistics of differential metabolites (amino acids, carbohydrates, phospholipids, neurotransmitters, etc.); (**E**): Functional classification statistics of differential metabolites (environmental information processing, genetic information processing, metabolism, etc.); (**F**): KEGG pathway analysis of the enrichment of differential metabolites (bubble chart, showing significantly enriched metabolic pathways and *p*-values). CG: Control group; EG: *Eucommia ulmoides* leaf–*Astragalus membranaceus* complex group; QC: Quality control group.

Based on the growth performance, serum biochemical parameters, antioxidant status, immune responses, and gut microbiota results, the 42-day samples were selected for serum metabolomic analysis to further investigate the potential metabolic mechanisms underlying the long-term regulatory effects of EG supplementation. Serum metabolomics identified a total of 1233 metabolites in the CG and EG groups, with 18 metabolites uniquely detected in the CG group and 28 metabolites uniquely detected in the EG group (Figure 7A). PCA demonstrated clear separation between CG and EG groups, indicating that dietary EG supplementation induced substantial alterations in the serum metabolic profile of yellow-feathered broilers (Figure 7B). Using VIP > 1 and *p* < 0.05 as screening criteria, a total of 103 differential metabolites were identified, including 77 up-regulated and 26 down-regulated metabolites (Figure 7C). KEGG enrichment analysis revealed that these differential metabolites were mainly involved in six metabolic pathways, including the GnRH signaling pathway, glycine, serine and threonine metabolism, glycerophospholipid metabolism, alanine, aspartate and glutamate metabolism, arginine biosynthesis, and pantothenate and CoA biosynthesis. Among these metabolites, Lysopc (15:0), panthenol, argininosuccinic acid, and L-aspartic acid were significantly up-regulated, whereas 5-aminolevulinic acid and Lpe (16:0) were significantly down-regulated in the EG group (Figure 7D–F). Collectively, these findings indicate that long-term dietary EG supplementation promotes metabolic remodeling in yellow-feathered broilers, particularly through regulation of amino acid, lipid, and energy metabolism pathways, which may contribute to the improvements in growth performance and physiological homeostasis.

### 3.9. Correlation Analysis Between the Gut Microbiota and Differential Serum Metabolites

**Figure 8:**
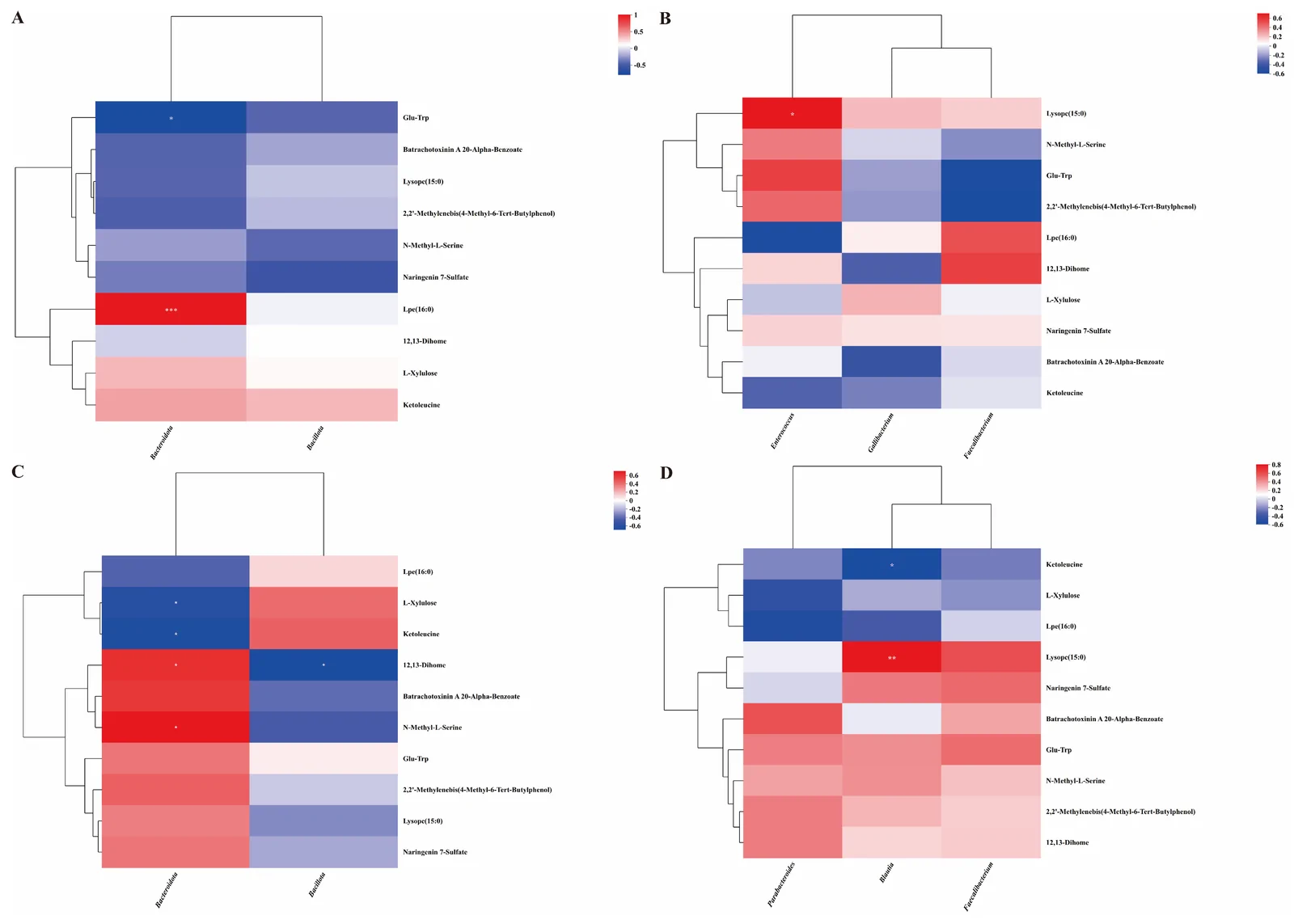
Spearman’s correlation heatmaps between the gut microbiota and differential serum metabolites in yellow-feathered broilers. Note: (**A**) Correlations between jejunal bacterial phyla and differential serum metabolites; (**B**) correlations between jejunal bacterial genera and differential serum metabolites; (**C**) correlations between cecal bacterial phyla and differential serum metabolites; and (**D**) correlations between cecal bacterial genera and differential serum metabolites. Red indicates a positive correlation, whereas blue indicates a negative correlation; color intensity represents the magnitude of the correlation coefficient. Asterisks indicate statistical significance: * *p* < 0.05, ** *p* < 0.01 and *** *p* < 0.001. The dendrograms for the rows and columns represent hierarchical clustering based on the correlation patterns.

To further investigate the potential associations between gut microbial composition and alterations in host serum metabolism, Spearman’s correlation analysis was performed to evaluate the relationships between the major phyla and genera in the jejunum and cecum and differential serum metabolites (Figure 8). At the jejunal phylum level, *Bacteroidota* was negatively correlated with Gln–Trp (*p* < 0.05) but positively correlated with Lpe (16:0) (*p* < 0.001; Figure 8A). At the jejunal genus level, Enterococcus was positively correlated with Lysopc (15:0) (*p* < 0.05; Figure 8B). At the cecal phylum level, *Bacteroidota* was negatively correlated with L-xylulose and ketoleucine but positively correlated with 12,13-DiHOME and N-methyl-L-serine (*p* < 0.05); *Bacillota* was negatively correlated with 12,13-DiHOME (*p* < 0.05; Figure 8C). At the cecal genus level, *Blautia* was negatively correlated with ketoleucine (*p* < 0.05) but positively correlated with Lysopc (15:0) (*p* < 0.01; Figure 8D). Other microbial taxa also exhibited varying positive or negative correlations with the selected metabolites, although these correlations did not reach statistical significance. Overall, significant associations were identified between the jejunal and cecal microbiota and several differential serum metabolites, suggesting a potential biological link between EG-associated changes in gut microbial composition and alterations in host systemic metabolism.

## 4. Discussion

This study systematically evaluated the effects of dietary supplementation with the *Eucommia ulmoides* leaf–*Astragalus membranaceus* complex in yellow-feathered broilers by integrating compositional characterization, in vitro antioxidant activity, growth performance, serum parameters, gut microbiota, and serum metabolomics. EG contained multiple plant-derived bioactive constituents and exhibited concentration-dependent free-radical-scavenging activity in vitro. Among the inclusion levels tested, EG (0.5%) produced the most consistent growth-promoting effects, as evidenced by increased body weight and average daily gain and a reduced feed conversion ratio, without significantly affecting feed intake. These findings suggest that EG primarily improved feed utilization efficiency rather than stimulating feed consumption. In contrast, higher inclusion levels provided no additional benefits, which may have been partly associated with their potential effects on dietary palatability. Therefore, EG (0.5%) may provide an appropriate balance between the effective supply of bioactive constituents and dietary palatability. EG supplementation also increased the activities of certain serum antioxidant enzymes and immunoglobulin concentrations, with the EG (0.5%) inclusion level generally producing the most consistent responses at both sampling time points, whereas routine serum biochemical parameters remained stable. In addition, alterations in the gut microbial composition and serum metabolite profile were observed in the EG (0.5%) group, providing biological insights into the potential association between EG supplementation and improved feed utilization efficiency.

The biological activity of phytogenic feed additives is closely related to the identity, concentration, and interactions of their bioactive constituents [30]. Chemical characterization showed that EG contained polysaccharides, flavonoids, saponins, chlorogenic acid, and calycosin-7-O-β-D-glucoside, with polysaccharides representing the most abundant active fraction. Dietary plant polysaccharides have been reported to regulate antioxidant capacity and immune responses in broilers and may therefore constitute an important functional basis for phytogenic feed additives [31]. Flavonoids can also modulate antioxidant and immune-related processes in broilers [32], whereas dietary saponins have been associated with the regulation of immune responses, intestinal integrity, and nutrient transport under enteric challenge conditions [33]. In complex phytogenic preparations, these compound classes may collectively contribute to the overall biological activity through multiple and potentially interacting mechanisms [30]. However, the respective contributions and specific interactions of these constituents within EG were not directly evaluated in the present study. Collectively, the identified constituents establish the compositional basis of EG and provide a chemical context for interpreting its in vitro activity and the biological responses observed in vivo. In vitro assays showed that EG scavenged DPPH, ABTS, and hydroxyl radicals in a concentration-dependent manner and exhibited Fe^3+^-reducing capacity. Although its IC_50_ values were higher than those of vitamin C, indicating lower radical-scavenging potency than the positive control, these findings confirm the chemical antioxidant activity of EG. Chlorogenic acid can participate in radical scavenging by donating hydrogen atoms and inhibiting lipid peroxidation, while the phenolic hydroxyl groups of flavonoids can stabilize radical intermediates [34,35]. Polysaccharides derived from *A. membranaceus* and *E. ulmoides* have also been reported to scavenge reactive oxygen species and influence endogenous antioxidant systems [36,37]. The in vitro antioxidant activity of EG may therefore arise from the combined actions of several phytochemical constituents. Nevertheless, antioxidant activity measured in a cell-free system and systemic antioxidant responses in animals represent different levels of evidence and do not necessarily exhibit concordant responses.

SOD and GSH-Px are important components of the enzymatic antioxidant defense system that remove reactive oxygen species and maintain redox homeostasis [5,38]. In the present study, all EG-supplemented groups showed significantly higher serum SOD activity than the control group at 21 days of age, whereas GSH-Px activity was unchanged. At 42 days of age, both SOD and GSH-Px activities were significantly increased by all three EG inclusion levels. The EG (0.5%) group generally exhibited the greatest numerical activities and had significantly higher SOD activity at 21 days and GSH-Px activity at 42 days than the 2.0% group. These findings indicate that the in vivo antioxidant response to EG was dependent on the enzyme measured and the duration of supplementation rather than following a simple linear dose–response pattern. The delayed increase in GSH-Px activity may be associated with longer exposure to the bioactive constituents of EG or with age-related changes in redox metabolism, although these possibilities were not directly examined. Together with its concentration-dependent radical-scavenging activity in vitro, the increases in serum antioxidant enzyme activities support the antioxidant potential of EG in vivo. Nevertheless, measurements of SOD and GSH-Px alone do not establish an overall reduction in oxidative damage. Future studies incorporating markers such as malondialdehyde, total antioxidant capacity, and reactive oxygen species, as well as appropriate oxidative-stress models, are required to characterize the underlying redox mechanisms more comprehensively.

Serum IgA, IgM, and IgG are commonly used indicators of humoral immune status and reflect different aspects of mucosal immunity, primary immune responses, and secondary immune responses, respectively [39]. At 21 days of age, the 0.5% and 2.0% EG groups had significantly higher serum IgA and IgG concentrations than the control group, whereas IgM concentration was increased by all three inclusion levels. At 42 days of age, all EG-treated groups showed higher IgA and IgM concentrations than the control group, while a significant increase in IgG was detected only in the EG (0.5%) group. These results suggest that EG enhanced selected humoral immune-related indices, but the response varied among immunoglobulin classes, ages, and inclusion levels. The generally stronger and more persistent responses in the EG (0.5%) group, together with the absence of additional improvement at 1.0% or and 2.0%, further indicate a nonlinear supplementation response. Previous studies have shown that *A. membranaceus polysaccharides* can affect immune-cell activity and immune-organ function, whereas chlorogenic acid has been associated with anti-inflammatory activity and the maintenance of immune homeostasis [9,40,41]. These findings provide a biological basis for the immunoglobulin responses observed in the present study and suggest that polysaccharides, phenolic compounds, and other constituents in EG may have acted individually or cooperatively. However, circulating immunoglobulin concentrations represent only one component of the immune response and do not, by themselves, demonstrate enhanced resistance to infection. Further studies using immune-challenge models and incorporating cytokines, immune-organ indices, mucosal immune markers, and cellular immune responses are required to clarify the mechanisms and functional significance of the immunomodulatory effects associated with EG.

Consistent with the changes in serum antioxidant and immune-related indices, growth performance also responded to EG supplementation. Dietary inclusion of EG (0.5%) increased body weight and average daily gain and reduced the feed conversion ratio during both the 1–21 and 21–42 day phases, whereas feed intake remained unchanged. The 2.0% treatment reduced the feed conversion ratio only during the starter phase, while the remaining responses in the 1.0% and 2.0% groups were generally intermediate. Previous studies have similarly shown that *A. membranaceus* based feed additives can increase average daily gain and reduce the feed conversion ratio in broilers without increasing feed intake [13,42]. *A. membranaceus polysaccharides* may improve growth and feed utilization by influencing intestinal function and nutrient metabolism [9,43], whereas *E. ulmoides* leaf extracts have been reported to improve growth and oxidative status in broilers and to alter gut microbial and serum metabolic profiles in laying hens [17,18]. The unchanged feed intake in the present study supports the interpretation that the growth response to EG was associated primarily with improved feed utilization rather than appetite stimulation, in agreement with previous findings concerning plant-derived bioactive compounds [30,44]. However, because nutrient digestibility, digestive enzyme activity, and intestinal morphology were not directly measured, these pathways should be regarded as priorities for mechanistic validation rather than established explanations.

The growth-performance results were not consistent with a monotonic dose–response pattern. Among the three inclusion levels, EG (0.5%) most consistently increased body weight and average daily gain and reduced the feed conversion ratio across both growth phases, whereas increasing the inclusion level to 1.0% and 2.0% did not provide sustained additional benefits. The EG (0.5%) group also generally showed the strongest and most persistent increases in serum SOD, GSH-Px, IgA, IgM, and IgG across the two sampling times, while the higher inclusion levels produced weaker or less consistent responses. Thus, EG (0.5%) represented the most consistently effective and biologically representative level among the doses tested, while 1.0% and 2.0% provided no additional overall benefit. Because doses below EG (0.5%) were not evaluated and a formal dose–response model was not fitted, EG (0.5%) cannot yet be defined as the minimum effective or biologically optimal dose. Nevertheless, it provides a rational starting point for further dose optimization and application-oriented validation.

Serum biochemical indices provide information on nutrient metabolism, electrolyte balance, and organ-related physiological status. Total protein, albumin, and globulin are commonly used to characterize circulating protein status; phosphorus, Ca^2+^, Na^+^, K^+^, and Cl^−^ contribute to mineral and electrolyte homeostasis; and uric acid is a major end product of purine and nitrogen metabolism in poultry [45]. At both 21 and 42 days of age, EG did not significantly affect AST, ALB, GLU, CPK, TP, PHOS, UA, BA, GLOB, Na^+^, K^+^, Ca^2+^, Cl^−^, the A/G ratio, corrected calcium, and the Na^+^/K^+^ ratio. The comparability of these parameters among treatments indicates that EG did not significantly alter the measured indicators associated with protein metabolism or mineral and electrolyte homeostasis under the present experimental conditions. Therefore, EG did not induce detectable serum biochemical abnormalities at the inclusion levels and during the feeding period evaluated, providing preliminary evidence supporting its feeding safety in yellow-feathered broilers. Moreover, the improved growth performance observed in the EG (0.5%) group was not accompanied by significant changes in these routine serum biochemical parameters, suggesting that the underlying mechanisms may involve nutrient digestion and utilization, intestinal microbial ecology, and molecular metabolism rather than marked alterations in routine systemic biochemical status.

The gut microbiota contributes to nutrient transformation, energy harvest, immune interactions, and maintenance of the intestinal barrier and is closely associated with poultry physiology [46,47]. EG did not significantly alter the Chao1, ACE, Shannon, and Simpson indices in the jejunum and cecum at either 21 and 42 days of age, indicating that α-diversity remained broadly comparable among groups. In contrast, the numbers of shared and unique OTUs in the Venn diagrams and the separation observed in PLS-DA suggested that microbial community composition may have differed among treatments. Microbial diversity describes community richness and evenness, whereas community composition concerns the identity and relative abundance of individual taxa; consequently, changes in specific taxa do not necessarily coincide with altered α-diversity. At the phylum level, the intestinal microbiota was dominated by *Bacillota* and *Bacteroidota*, members of which participate in carbohydrate fermentation, short-chain fatty acid production, and energy metabolism [7,48]. Several phyla displayed different relative-abundance patterns across intestinal segments and sampling times following EG supplementation, demonstrating that taxon-specific analyses can provide information beyond that captured by α-diversity indices. The responses at 21 and 42 days were not fully consistent. This temporal variation may reflect age-related microbial maturation, transition between feeding phases, changes in fermentable-substrate availability, and longer exposure to EG; however, these explanations remain to be tested. The stage-dependent patterns observed here highlight the importance of distinguishing the effects of age, dietary phase, and additive exposure in future experimental and statistical designs. At the genus level, the relative abundances of *Faecalibacterium* and *Blautia* in the cecum were higher in the EG group at day 21, whereas *Faecalibacterium* and *Parabacteroides* were the principal genera showing higher relative abundance at day 42. *Faecalibacterium* is a recognized butyrate-producing genus associated with energy supply to intestinal epithelial cells and maintenance of barrier function [48,49]. Lower relative abundances of *Enterococcus* and *Acinetobacter* were observed in the EG group at some sampling points. Conversely, *Escherichia* and *Gallibacterium* showed higher relative abundances in the jejunum at certain sampling points, indicating that the microbial response to EG was selective and stage- and site-dependent rather than uniformly favorable. The directional changes in *Faecalibacterium* and *Blautia* are biologically consistent with previous observations that plant polysaccharides and phenolic compounds can influence short-chain-fatty-acid-associated taxa by supplying fermentable substrates and modifying the intestinal environment [29]. They are also compatible with the known potential of *A*. *membranaceus* polysaccharides and *E. ulmoides* phytochemicals to modulate the intestinal milieu. This consistency supports the biological plausibility of the findings but does not demonstrate that specific EG constituents directly caused the observed microbial changes [9,10,18]. Nevertheless, these genera represent useful candidates for future studies examining the relationship between EG supplementation and intestinal nutrient transformation, particularly when combined with measurements of short-chain fatty acids and microbial functional genes.

Metabolomics enables systematic detection of small-molecule responses to dietary interventions and facilitates identification of candidate metabolic processes [50]. At 42 days of age, 1233 serum metabolites were detected, of which 103 met the predefined criteria for differential abundance, including 77 upregulated and 26 downregulated metabolites. Separation between the CG and EG (0.5%) groups in the PCA also indicated distinguishable serum metabolic profiles. Together with the increased serum SOD and GSH-Px activities and immunoglobulin concentrations following EG supplementation, these findings indicate that EG was associated with concurrent systemic antioxidant, immune, and metabolic responses. KEGG enrichment analysis mainly implicated pathways related to amino acid, glycerophospholipid, and energy metabolism, as well as endocrine processes. In addition to its reproductive role, GnRH signaling has been linked to growth, immunity, and metabolic regulation [51]. Nevertheless, its specific biological relevance in the yellow-feathered broilers examined here requires validation using targeted molecular endpoints. More generally, pathway enrichment identifies pathways that are statistically over-represented among the differential metabolites; however, such findings should be interpreted as candidate biological processes potentially affected by EG supplementation rather than direct evidence of pathway activation [52]. The amino-acid-related pathways are particularly relevant because glycine, serine, threonine, alanine, aspartate, glutamate, and arginine participate in protein synthesis, nitrogen metabolism, anaplerotic reactions of the tricarboxylic acid cycle, and energy production [53]. Therefore, the increased abundances of argininosuccinic acid and L-aspartic acid may reflect alterations in nitrogen transfer and energy-substrate utilization, potentially contributing to the increased average daily gain and reduced feed conversion ratio observed in the EG (0.5%) group while providing metabolic support for antioxidant defense and immunoglobulin synthesis. Regarding glycerophospholipid metabolism, Lysopc (15:0) was increased, whereas Lpe (16:0) was decreased following EG supplementation. Lysopc (15:0) participates in lipid transport, membrane remodeling, and cellular signaling, whereas phosphatidic acid is an important intermediate in lipid biosynthesis and signaling [54,55]. These changes may reflect adaptations in lipid transport, membrane turnover, and immune-related cellular signaling. The decrease in 5-aminolevulinic acid may also be related to heme biosynthesis and mitochondrial metabolism [56], although its functional significance remains unclear. These metabolic alterations were biologically consistent with the increased serum SOD and GSH-Px activities observed at 42 days of age. Enrichment of pantothenate and coenzyme A biosynthesis, together with the increased abundance of panthenol, further suggests alterations in energy-substrate utilization. Coenzyme A links carbohydrate, lipid, and amino acid metabolism [57,58]; therefore, changes in this pathway may help explain the improved feed conversion ratio and the metabolic demands associated with enhanced antioxidant and humoral immune responses. Spearman’s correlation analysis further linked the microbiome and metabolome findings: *Bacteroidota* was associated with Gln–Trp, N-methyl-L-serine, ketoleucine, L-xylulose, Lpe (16:0), and 12,13-DiHOME; *Bacillota* was associated with 12,13-DiHOME; and *Enterococcus* and *Blautia* were associated with Lysopc (15:0). These associations suggest that EG-related alterations in intestinal microbial composition and systemic metabolism were biologically interconnected and consistent with the improvements in feed utilization, antioxidant enzyme activities, and immunoglobulin concentrations, particularly at the 0.5% inclusion level.

Collectively, this study provides a multilevel framework encompassing the chemical composition and in vitro activity of EG, broiler growth phenotypes, serum indices, intestinal microbial composition, and serum metabolic responses. Within the tested range, EG (0.5%) consistently improved growth and feed utilization, supporting its value for further dose optimization and application-oriented evaluation. The concurrent increases in selected serum antioxidant enzyme activities and immunoglobulin concentrations indicate that the response extended beyond growth performance, whereas the absence of significant changes in routine serum biochemical indices suggests that the measured markers of protein metabolism, organ-related status, and electrolyte balance remained stable under the present conditions. The observed differences in microbial composition and serum metabolite profiles provide complementary clues that EG may influence intestinal nutrient transformation and host substrate utilization.

## 5. Conclusions

Dietary supplementation with the *Eucommia ulmoides* leaf–*Astragalus membranaceus* complex improved the growth performance of yellow-feathered broilers. Among the tested inclusion levels, EG (0.5%) produced the most consistent increases in BW and ADG and reductions in FCR without affecting feed intake, whereas increasing the inclusion level to 1.0% and 2.0% provided no additional benefit. EG supplementation also enhanced selected serum antioxidant and humoral immune indices. Meanwhile, the unchanged serum biochemical profile indicated that protein metabolism and mineral and electrolyte balance were not detectably altered under the present experimental conditions. At the 0.5% inclusion level, EG reshaped intestinal microbial composition without significantly affecting alpha diversity, with enrichment of potentially beneficial cecal genera, including *Faecalibacterium*, *Blautia*, and *Parabacteroides*. Serum metabolomics further revealed changes in metabolites and pathways associated primarily with amino acid, glycerophospholipid, and energy metabolism. These microbial and metabolic changes provide a reasonable biological link for improved growth and feed utilization. The observed effects may be related to the comprehensive biological activities of EG components, including polysaccharides, flavonoids, saponins, chlorogenic acid, and calycosin-7-O-β-D-glucoside. Overall, within the tested range, EG (0.5%) is the most effective addition level and is a promising plant-derived feed additive that helps improve growth efficiency and supports the physiological health of yellow-feathered broilers.

## Figures and Tables

**Table 1:** Composition and nutrient levels of basal diets for yellow-feathered broilers.

Ingredient Composition of Diets (%)		
Ingredient	Starter Diet (0–3 Weeks; 881)	Grower-Finisher Diet (4–6 Weeks; 882)
Corn	61.32	66.08
Soybean meal (46% CP)	21.20	15.10
Cottonseed meal (46% CP)	3.00	3.00
Corn gluten meal (60% CP)	4.80	4.50
Nucleotide by-product (70%)	2.00	2.00
Soybean oil	3.00	5.00
Limestone	1.47	
Limestone (coarse)		1.35
Dicalcium phosphate	1.10	0.96
Sodium bicarbonate	0.10	
Sodium chloride	0.25	0.35
Lysine (98%)	0.36	0.32
Methionine (99%)	0.20	0.14
Premix	1.20	1.20
**Total**	**100**	**100**
**Nutrient Composition of Diets**		
**Nutrient**	**Starter Diet (0–3 Weeks)**	**Grower-Finisher Diet (4–6 Weeks)**
Metabolizable energy (kcal/kg)	3040.00	3200.00
Crude protein (%)	20.80	18.10
Calcium (%)	0.90	0.81
Total phosphorus (%)	0.55	0.50
Available phosphorus (%)	0.28	0.25
Sodium (%)	0.14	0.15
Chlorine (%)	0.19	0.24
Lysine (%)	1.20	1.00
Methionine (%)	0.55	0.46

**Table 2:** Nutritional composition and energy level of *Eucommia ulmoides* leaf-*Astragalus membranaceus* complex.

Components	As Sampled Basis	Dry Matter Basis
Moisture/%	6.00	
Dry Matter/%	94.00	
Crude Protein/%	20.12	21.40
ADICP/%	3.67	3.90
Available Protein/%	16.45	17.50
SP%CP/%		29.00
NDICP/%	3.85	4.10
ADF/%	31.96	34.00
NDF/%	34.59	36.80
Lingnin/%	2.16	2.30
NFC/%	27.96	29.74
Starch/%	8.46	9.00
Fat/%	2.41	2.56
Ash/%	8.93	9.50
TDN/%		69.00
NEM, Mcal/kg	1.48	1.57
NEG, Mcal/kg	0.91	0.97
Calcium/%	1.06	1.13
Phosphorus/%	0.39	0.41
Magnesium/%	0.45	0.48
Potassium/%	2.57	2.73
Sulfur/%	0.19	0.20
Chloride/%	0.01	0.01
Ca/P		2.76
RDP-N/S		11.81
K/Mg		5.69
TFA/%	0.01	0.01

Note: ADICP, Acid Detergent Insoluble Crude Protein; SP%CP, Soluble Protein as % of Crude Protein; NDICP, Neutral Detergent Insoluble Crude Protein; ADF, Acid Detergent Fiber; NDF, Neutral Detergent Fiber; NFC, Non-Fiber Carbohydrate; TDN, Total Digestible Nutrients; NEM, Mcal/kg, Net Energy for Maintenance; NEG, Mcal/kg Net Energy for Gain; Ca/P, Calcium to Phosphorus Ratio; K/Mg, Potassium to Magnesium Ratio; RDP-N/S, Rumen Degradable Protein Nitrogen/Sulfur Ratio.

**Table 3:** Effects of *Eucommia ulmoides* leaf-*Astragalus membranaceus* complex on growth performance of broilers during 1–21 d and 21–42 d.

Item	CG	EG (0.5%)	EG (1%)	EG (2%)
**BW 1 d (g)**	41.79 ± 0.98	41.58 ± 0.88	41.20 ± 0.85	41.66 ± 0.77
**BW 21 d (g)**	315.00 ± 27.39 ^b^	347.00 ± 20.19 ^a^	336.00 ± 16.73 ^ab^	335.00 ± 15.81 ^ab^
**ADG 1–21 d (g/d)**	13.01 ± 1.32 ^b^	14.54 ± 0.93 ^a^	14.04 ± 0.80 ^ab^	13.97 ± 0.72 ^ab^
**FI 1–21 d (g)**	23.95 ± 1.94	24.52 ± 0.81	25.10 ± 1.10	23.71 ± 0.88
**FCR 1–21 d**	1.84 ± 0.05 ^a^	1.69 ± 0.07 ^b^	1.79 ± 0.08 ^ab^	1.70 ± 0.08 ^b^
**BW 42 d (g)**	1004.44 ± 36.73 ^d^	1093.33 ± 24.97 ^a^	1041.11 ± 28.76 ^bcd^	1028.89 ± 18.59 ^cd^
**ADG 21–42 d (g/d)**	32.75 ± 1.92 ^b^	35.50 ± 0.80 ^a^	33.56 ± 0.99 ^ab^	33.02 ± 1.49 ^b^
**FI 21–42 d (g)**	74.76 ± 4.92	76.67 ± 2.78	75.03 ± 1.76	73.70 ± 3.20
**FCR 21–42 d**	2.28 ± 0.04 ^a^	2.16 ± 0.08 ^b^	2.24 ± 0.02 ^ab^	2.23 ± 0.02 ^ab^

Note: BW 1 d, body weight at 1 day; BW 21 d, body weight at 21 days; BW 42 d, body weight at 42 days; ADG, average daily gain; FCR, feed conversion ratio; FI, feed intake. Values are expressed as mean ± SE. Different superscript letters within the same row indicate significant differences among treatments (*p* < 0.05).

## Data Availability

The original contributions presented in this study are included in the article. Further inquiries can be directed to the corresponding author.
